# Chemokine Coreceptor-2 Gene Polymorphisms among HIV-1 Infected Individuals in Kenya

**DOI:** 10.1155/2015/952067

**Published:** 2015-08-02

**Authors:** Dorcas Wachira, Raphael Lihana, Vincent Okoth, Alex Maiyo, Samoel Ashimosi Khamadi

**Affiliations:** Kenya Medical Research Institute, Nairobi 54628, Kenya

## Abstract

Chemokine Coreceptor-2 (CCR2) is an entry coreceptor for HIV-1. A mutation in the coding gene for this coreceptor, CCR2-64I, has been shown to be an important factor for delaying disease progression. In Kenya no studies have been done to determine the status of CCR2 gene polymorphisms among HIV-1 infected individuals. To determine the existence and distribution of CCR2 gene mutations and identify polymorphic groups of the coreceptor gene in the population, a cross-sectional study was conducted to analyze the differences in allelic frequencies of CCR2-64I among HIV-1 seropositive individuals. Blood samples were collected from HIV/AIDS screening centers and analyzed for the presence of CCR2-64I using restriction fragment length polymorphism (RFLP). One hundred and eighteen samples collected from different regions of the country were genotyped for the CCR2-64I mutation. Of these, 4 (3.4%) were homozygous mutants (I/I) and 21 (17.8%) were heterozygous (V/I). Ninety-three subjects (78.8%) were wild type (V/V). With the search for a preventive/therapeutic HIV vaccine elusive, the presence of CCR-2 gene polymorphisms that delay disease progression and prolong the lives of the infected in the Kenyan population may contribute to the growing evidence that host genetic factors are important in predicting susceptibility to HIV-1 infection.

## 1. Introduction

The natural history and pathogeneses of the human immunodeficiency virus type 1 (HIV-1) infections are characterized by many viral and host factors and their interactions. First, entry of HIV-1 into target cells requires both CD4 and one of the chemokine receptors. Two chemokine receptors, chemokine (C–C motif) receptor 5 (CCR5) and chemokine (C–X–C motif) receptor 4 (CXCR4), are the major coreceptors for macrophage-cell-tropic (M-tropic or R5-tropic) and T-cell-tropic (T tropic or X4-tropic) HIV-1 infection, respectively [1]. Other chemokine receptors, CCR2, CCR3, CCR8, CCR9, CX(3)CR1, CXCL12 (SDF1), and CCL5 (RANTES), can act as alternative coreceptors for mediating some strains of HIV-1 infection. Genetic variants of these genes, which encode HIV-1 coreceptors and their ligands, have been implicated in the susceptibility to HIV-1 infection, and their prevalence varies by ethnicity.

Polymorphisms of the genes for CCR5 (CCR5-Delta32), CCR2, and stromal-derived factor 1 (SDF1) have been found to modulate the susceptibility of individuals to HIV-1 infection and/or the pathogenic progression [2]. The CCR5Δ32 polymorphism encoding a 32-base pair deletion in the CCR5 coding region has been shown to result in a nonfunctional truncated protein [2]. Multiple studies have confirmed the protective impact of CCR5Δ32 on HIV disease and have identified additional CCR5 polymorphisms that alter disease progression [3, 4].

The CCR2 allele is a prominent receptor for the monocyte chemoattractant protein (MCP) group of C–C chemokines and is among the most important genetic factors known to be associated with host resistance to HIV-1 infection. A point mutation in the CCR2 gene that leads to a single, conservative amino acid change, which substitutes isoleucine for valine at position 64 (CCR2-64I), in the first transmembrane domain of CCR2 has been found to correlate significantly with delayed progression to acquired immunodeficiency syndrome (AIDS) [5].

Studies have also shown that homozygosity for this mutation is associated with a delayed progression to disease, while individuals who are heterozygous for CCR2-64I progress quickly to AIDS [6, 7]. Thus, polymorphisms in chemokine ligands and receptors may further impact disease acquisition and progression by mechanisms beyond viral entry.

The frequency of the CCR2-64I mutation varies in different ethnic populations. Several studies have shown that the CCR2-64I allele is broadly distributed in humans. The frequency of this polymorphism was 50% in the HIV-infected but long-term nonprogressors (LTNPs) Chinese but only 23% in typical progressors. This suggests that the high frequency of CCR2-64I allele is associated with a slower progression to AIDS [7].

In spite of the importance of chemokine receptors in AIDS pathogenesis, little information is available on the frequency of chemokine receptor gene polymorphisms modulating HIV-1 infection in the Kenyan population. This study was carried out to identify variants of the CCR2 gene among HIV/AIDS clients as the frequency of these genetic variants in Kenya, particularly in HIV-1 seropositive and/or seronegative high-risk populations, is currently unknown. Understanding how these genetic variants contribute to susceptibility to HIV-1 infection and disease progression could add to the existing knowledge on HIV pathogenesis.

## 2. Materials and Methods

### 2.1. Study Samples

After receiving informed consent from the clients, a total of 118 blood samples from HIV-1 infected participants in 8 provincial and district hospitals were collected in EDTA tubes. Five mL of blood was drawn from each client. Demographic data such as age, gender, and residence were obtained using a self-reporting questionnaire. The research work was approved by the Graduate School Board of Kenyatta University (reference I56/13034/05). The study was conducted according to the national and international regulations governing the use of human subjects in biomedical research.

### 2.2. Laboratory Procedures

Peripheral blood mononuclear cells (PBMCs) were obtained from 5 mL of whole blood by density gradient centrifugation using Ficoll-Paque Plus as per the manufacturer's guidance. Briefly, whole blood was added to 15 mL of PBS buffer ph 7.2 containing 2 mM EDTA and chilled to 2–8°C. This mixture was layered carefully over 15 mL of Ficoll-Paque in a 50 mL falcon tube. This was centrifuged at 400 ×g for 30 minutes at 20°C in a swinging-bucket rotor without brakes. The upper layer was aspirated leaving the PBMC mononuclear cell layer undisturbed. This layer was transferred to a new tube and mixed with the PBS buffer mentioned above and span at 300 ×g for 10 minutes to clean the mononuclear cells. DNA was extracted from the cells using DNAzol and ethanol precipitation.

Amplification of a 128-base pair fragment of the CCR2 gene was carried out using CCR2 gene primers CKR2_1A (sense): 5′ TTG TGG GCA ACA TGA TGG and CKR2_1Z (antisense): 5′ GAG CCC ACA ATG GGA GAG TA in a reaction mixture containing 10 pmol of each primer, 2 *μ*L of extracted DNA, and a commercial PCR master mix. After an initial denaturation step at 94°C for 5 minutes, PCR was run for 40 cycles of 94°C for 1 minute, 55°C for 1 minute, and 72°C for 1 minute. The resulting amplicons were digested with* BsaBI* restriction enzyme at 60°C for 2 hours. The products were then electrophoresed on a 4% agarose gel and stained with ethidium bromide for visualization. A 128 bp fragment indicated a homozygous wild genotype, while 110 bp and 18 bp fragments were indicative of the homozygous mutant genotype. The presence of three fragments revealed the heterozygous genotype.

### 2.3. Statistical Analysis

Genotype frequencies were evaluated using the Hardy-Weinberg equilibrium test. Allele frequencies and the prevalence of genotypes were determined using the *χ* ^2^ test. Statistical significance was defined as *P* ≤ 0.05.

## 3. Results

A total of 118 samples were collected and analyzed. Among these, 4 (3.4%) had the CCR2-*V64I* homozygous mutation A/A while 21 (17.8%) had the heterozygous mutation G/A. Distribution of the wild type gene in the provinces ranged from 93.3% in Nairobi to 69.2% in Coast province. Nyanza province had the highest heterozygous mutations (25%) followed by Central, Coast, North Eastern, and Rift Valley provinces with all three having approximately 22%, while Nairobi province had the lowest (6.7%). A single case of homozygous mutations was present in only four provinces (Table 1). There were however no significant differences in the distribution of CCR2-V641 mutations across the provinces (*P* = 0.98). Within the provinces, there was a significant difference in the distribution of G/A mutations compared to A/A mutations (*P* < 0.001). Generally, the allelic genotypic frequency of the CCR2-V64I in all the eight provinces was below 8% of the samples analyzed.

## 4. Discussion

The prevalence of the mutant homozygous and heterozygous CCR2-64-I alleles was significant considering the small number of samples that were analyzed. This is as expected considering that mutations occur at low levels in populations. The significantly high CCR2-64-I mutations in the sampled population could be an indication that the mutation is common in the population. It could also suggest that this is an established mutation that has been passed down through generations even before the advent of the HIV/AIDS scourge. Similar studies have been carried out in other populations that show a significantly high distribution of the CCR2-64-I allelic mutations [8]. In a study in Moscow in 2001, for example, the wild type CCR2 gene alleles were 77.87% with the rest being CCR2-64-I heterozygotes without any CCR2-64-I mutant homozygotes [5]. In a similar study in Brazil in 2002, it was observed that the frequency of the CCR2 wild type genotype was 60% while that of the heterozygous mutant genotype was 44% while the homozygous mutant genotype was 3% [9]. In a similar study carried out in Kenya in 1998, it was observed that frequency of the CCR2-64I allele was 23% in a cohort of commercial sex workers [1]. This is comparable to what was seen in this study though there were variations within the different provinces of Kenya.

The CCR2-V64I mutation affects the gene that encodes the CCR2 receptor on the outside of cells and is more common than the CCR5 mutation; between 10 and 25% of the population are believed to have at least one mutant CCR2 gene [2]. In a study of over 3000 HIV-positive people, those who had one mutant CCR2 gene developed AIDS two to four years later than people who had two normal copies of the CCR2 gene. The results were even more striking when the data on the effects of CCR2 and CCR5 mutations were combined. About 30% of long-term survivors who had been infected with HIV for at least 16 years or more without developing AIDS had at least one CCR2 or CCR5 mutant gene [10]. The protective effect of CCR2B-64I has also been demonstrated by other studies [1].

## 5. Conclusions

This study survey focused on a relatively small number of individuals. However, the findings contribute to the growing evidence that the presence and effects of genetic variants that have been understudied in the African population are still important when predicting hosts susceptibility to HIV-1 and progression to AIDS within the sub-Saharan African population and more so in our Kenyan population. The knowledge of this mechanism of HIV entry into cells has resulted in the development of a new class of ARVs entry inhibitors, aimed at blocking the CCR5, CCR2, or CXCR4 coreceptors.

## Figures and Tables

**Table 1:** Summary of CCR2 gene polymorphisms in each of the 8 provinces of Kenya.

Province	Genotype			Total *N*
	CCR2	CCR2	CCR2	
	G/G (wt/wt)	G/A (wt/mut)	A/A (mut/mut)	
	*n* (%)	*n* (%)	*n* (%)	
Central	11 (78.6)	3 (21.4)	—	**14**
Coast	9 (69.2)	3 (23)	1 (7.7)	**13**
Eastern	13 (86.7)	2 (13.3)	—	**15**
Nairobi	14 (93.3)	1 (6.7)	—	**15**
North Eastern	11 (73.3)	3 (20)	1 (6.7)	**15**
Nyanza	12 (75)	4 (25)	—	**16**
Rift Valley	12 (75)	3 (18.8)	1 (6.3)	**16**
Western	11 (78.6)	2 (14.3)	1 (7.1)	**14**
**Total**	**93**	**21**	**4**	**118**
